# Peroral endoscopic myotomy: is it better to perform it in naive patients or as second-line therapy? Results of an open-label-controlled study in 105 patients

**DOI:** 10.1007/s00464-021-08767-6

**Published:** 2023-01-20

**Authors:** Raphael Olivier, Charlène Brochard, Stanislas Bruley des Varannes, Alain Ropert, Timothée Wallenhorst, Noémi Reboux, Lucille Quénéhervé, Emmanuel Coron

**Affiliations:** 1 Service de Gastroentérologie, CHU de Poitiers, Poitiers, France; 2 Institut des Maladies de l’Appareil Digestif, IMAD, CHU Nantes, Hôpital Hôtel-Dieu, 1 place Ricordeau, 44093 Nantes cedex, France; 3 Service des Maladies de l’Appareil Digestif, CHU Pontchaillou, Université de Rennes 1, Rennes, France; 4 Service d’Explorations Fonctionnelles Digestives, CHU Pontchaillou, Université de Rennes 1, Rennes, France; 5 CIC 1414, INPHY, Université de Rennes 1, Rennes, France; 6 Service de Gastroentérologie, CHRU de Brest, Brest, France; 7 Department of Gastroenterology and Hepatology, University Hospital of Geneva (HUG), rue Gabrielle Perret- Gentil 4, Genève, 1205-1211 Switzerland

**Keywords:** Achalasia, Endoscopy, Symptom score or index, Oesophagus

## Abstract

**Background:**

Whether Peroral Endoscopic Myotomy (POEM) can be proposed as a second-line treatment in patients with achalasia remains to be confirmed in real-life series.

**Objective:**

This study aimed to compare the efficacy, feasibility and safety of POEM between treatment-naïve patients and patients who had prior endoscopic or surgical therapies for achalasia.

**Methods:**

All consecutive patients who underwent a POEM procedure for achalasia in our centre from June 2015 to September 2018 were included in this retrospective study. They were classified into treatment-naïve patients (POEM1) and patients who had at least one previous endoscopic and/or surgical treatment for achalasia (POEM2).

**Results:**

A total of 105 patients were included, 52 in the POEM1 group and 53 in the POEM2 group. Clinical success (defined as an Eckardt score ≤ 3) at 6 months was observed in 93% of POEM1 patients and 84% of POEM2 patients (*p* = 0.18). Technical success rate was not significantly different between the two groups (100% vs 96%, respectively; *p* = 0.50). No significant difference was noted in terms of adverse event rate (19% vs 19%, respectively; *p* = 1.00). Post-procedure pain occurred in 12% of treatment-naive and 9% of non-naïve patients (*p* = 0.76). The median length of hospital stay was 3 days in both groups (*p* = 0.17). Symptomatic gastroesophageal reflux occurred in 25% of POEM1 patients and 16% of POEM2 patients (*p* = 0.24).

**Conclusion:**

Efficacy, feasibility and safety of POEM are not different between treatment-naïve and non-naïve patients. POEM is a valuable second-line approach in patients with persistent symptoms of achalasia after surgical or endoscopic treatments.

**Supplementary Information:**

The online version contains supplementary material available at 10.1007/s00464-021-08767-6.

Achalasia is a rare [1] primary motility disorder of the oesophagus, characterized by a loss of peristalsis of the oesophageal body associated with incomplete relaxation of the lower oesophageal sphincter (LES). Patients affected by this motility disorder can present with dysphagia, regurgitation and sometimes chest pain, associated with significant weight loss. Oesophageal manometry confirms the diagnosis and enables the classification of achalasia into three subtypes, which have different responses to treatment [2]. For many years, the therapeutic arsenal for achalasia was limited to pneumatic dilation, botulinum toxin injection and surgical Heller’s myotomy, which all show good short-term results with an efficacy of 77% [3], 83% [3] and 88% [4], respectively. However, 6 months after botulinum toxin injection, symptoms tend to recur and this treatment is therefore not recommended as a long-term solution by recent guidelines [3, 5]. Pneumatic dilation and Heller’s myotomy also carry a risk of recurrence at 5 years in up to 20% of patients [6, 7], requiring repeat Heller’s myotomy [8, 9] or pneumatic dilation [10, 11]. Since its description in 2010 by Inoue et al. [12], peroral endoscopic myotomy (POEM) has emerged as a novel approach for the endoscopic treatment of achalasia. Multiple studies have reported excellent clinical response in more than 80–90% of patients with a low rate of serious adverse events [13–16]. Initial management of achalasia is now based on shared decision-making between patients and physicians and takes into account manometric characteristics when choosing between pneumatic dilation, Heller’s myotomy or POEM [3, 5]. However, whether POEM should be proposed as a second-line approach in patients who relapse after prior therapy remains unclear. Indeed, although POEM has already been described as a rescue therapy for patients who relapse after Heller’s myotomy [17, 18], data concerning the efficacy and safety of POEM as a second-line treatment of symptomatic relapse of achalasia after Heller’s myotomy and/or endoscopic procedures (pneumatic dilation, botulinum toxin injection) are scarce [19]. Therefore, the aim of this study was to compare the efficacy, feasibility and safety of POEM between achalasia patients who had prior endoscopic or surgical therapy and those who did not receive any prior treatment.

## Materials and methods

### Study protocol

**Fig. 1:**
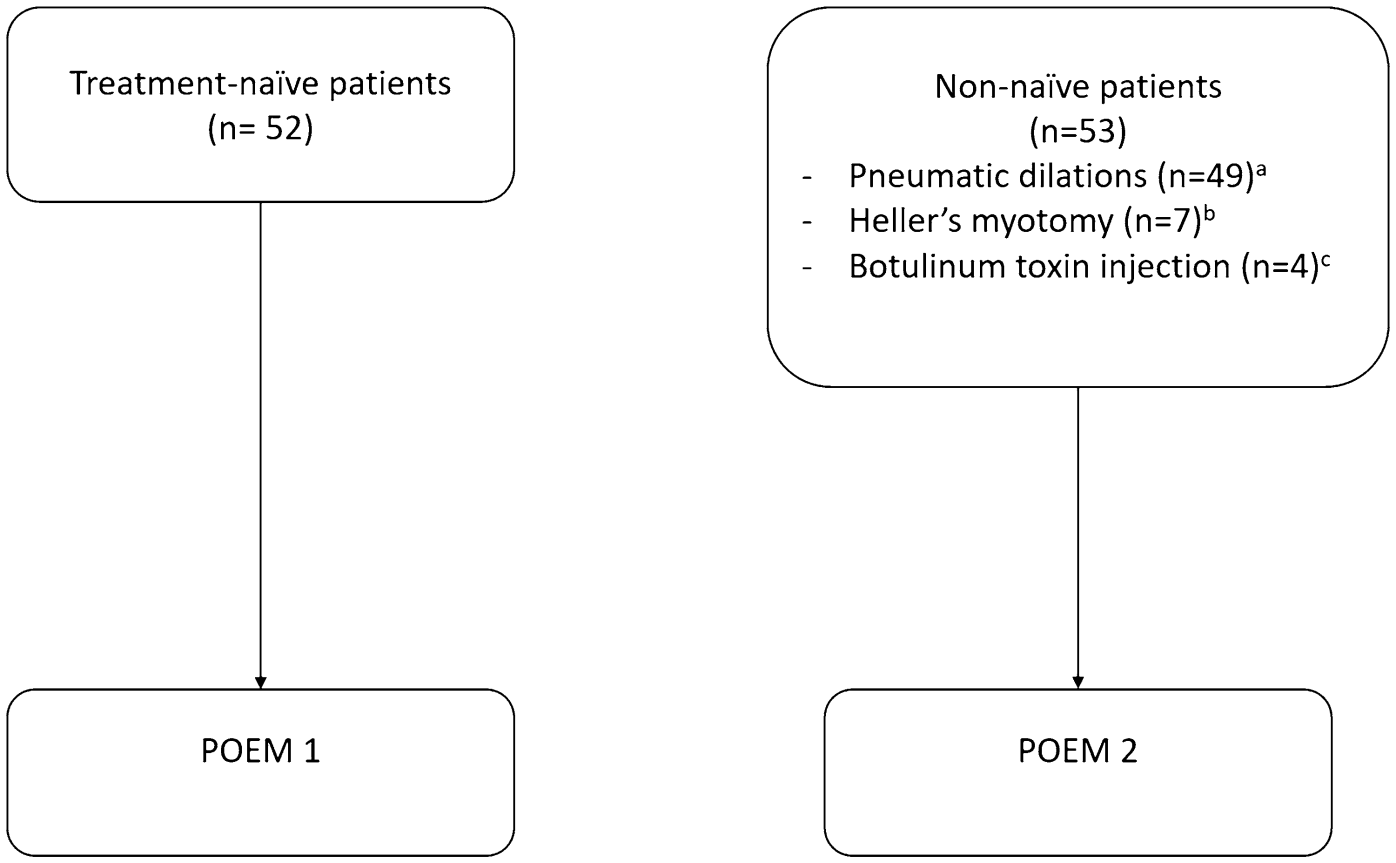
Flow-chart of the study. ^a^Mean number of dilations: 2.2. ^b^All patients except for one who underwent a Heller’s myotomy had been previously treated by pneumatic dilation. ^c^One patient who underwent botulinum toxin injection had also been treated by pneumatic dilation

All patients referred for POEM as a treatment of achalasia during a 3-year period (from June 2015 to September 2018) were included in this monocentric retrospective study carried out at a tertiary referral centre (University Hospital of Nantes, France) (Fig. 1). Informed consent of the procedure modalities and risks was obtained from each patient. Diagnosis of achalasia was based on high-resolution manometry findings and classified according to the Chicago classification of oesophageal motility disorders V3.0 [2].

Patients without any previous specific treatment of achalasia (naïve patients) were offered different options after full verbal information concerning classical therapies (botulinum toxin, endoscopic dilation or surgery) and POEM. The POEM1 group consisted of naïve patients currently treated by POEM as a first-line therapy. Another group of patients with achalasia was referred for POEM because of the recurrence of symptoms after receiving classical treatments. After providing information and clinical evaluation, the indication of POEM therapy was confirmed and the patients were included in the POEM2 group, excluding those with previous POEM history.

Patients with oesophageal cancer, history of oesophageal surgery (other than Heller’s myotomy) or with a follow-up of less than 6 months were excluded.

### Initial clinical evaluation

Pre-procedural evaluation included demographics (age, weight, height, percentage of weight loss), medical history (including cancer, autoimmune disease), medical treatment (including opioids), the type of oesophageal motility disorder, patient symptoms (the dominant symptom was differentiated), Eckardt score [20], duration of the disease and prior medical, endoscopic (pneumatic dilation, botulinum toxin injection) and/or surgical therapies (Heller’s myotomy). Manometric characterization included achalasia subtype determination, according to the Chicago classification using high-resolution manometry, resting pressure of the LES and integrated relaxation pressure (IRP). Upper GI endoscopy results were also recorded.

### POEM procedure

Procedural data collected included American Society of Anaesthesiologists (ASA) Physical Status Classification System score of the patient [21], date of procedure, full procedure (yes), length of myotomy, number of clips used to close the tunnel and the duration of the procedure.

**Fig. 2:**
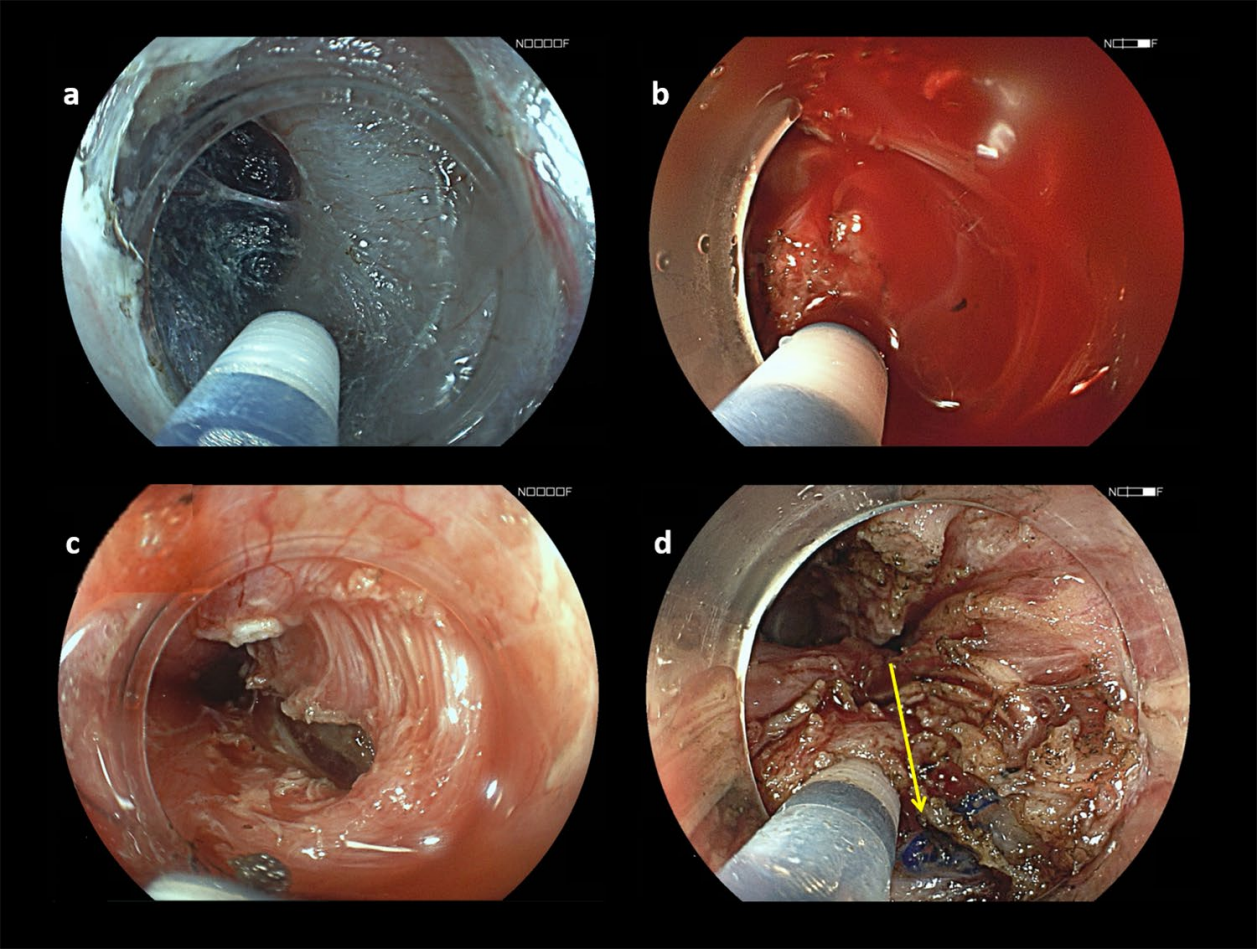
Endoscopic view of two POEM procedures. In a treatment-naïve patient, the submucosal space (**a**) and muscle (**c**) are non-fibrotic, thus allowing easy tunnelling and myotomy. In contrast, in a patient who previously underwent Heller’s myotomy, both submucosal tunnelling (**b**) and endoscopic myotomy (**d**) are more challenging. The arrow indicates surgical sutures from Heller’s myotomy (**d**)

All POEM procedures were performed by a single operator (EC) according to the original description of the technique [12] and were adapted as detailed below using an anterior approach. A high-definition gastroscope (EG760Z or EG590, Fujifilm, Japan) mounted with an endoscopic submucosal dissection (ESD) cap on the distal tip was used for all procedures. POEM procedures were conducted under general anaesthesia with endotracheal intubation and CO2 insufflation. Anticoagulant therapy and antiplatelet agents (other than aspirin) were discontinued before the procedure. The oesophagus was cleared of debris, food or saliva using endoscopic suction and/or a food retrieval basket. A submucosal injection was then carried out using a mix of saline and indigo carmine solution, 10 to 15 cm above the cardia. A transverse mucosal incision was performed using a second-generation ESD knife, allowing re-injection (Flushknife BT 1.5 mm, Fujifilm, Japan), and an electrosurgical generator (ERBE, Tübingen, Germany). The gastroscope was then introduced into the submucosal space and submucosal tunnelling was performed (Fig. 2). The tunnel was extended across the LES and into the gastric cardia. Both circular and longitudinal muscle layers were cut in order to provide a 10–12 cm long full-thickness myotomy using the same ESD knife. Closure of the mucosal incision was achieved using haemostatic clips (Olympus, Japan). Each case was recorded by video and still pictures. A water-soluble esophagogram was obtained the following day before initiating a liquid and soft diet. Patients were discharged home on day 2 or 3 if they were able to tolerate a soft diet without significant pain.

### Follow-up and outcome measures

All patients were systematically followed for 6 months after the procedure.

Post-procedural data included length of hospital stay, delayed adverse events, symptoms and Eckardt score, symptoms of gastro-oesophageal reflux disease (GERD), medication (e.g. proton pump inhibitors) at 3 and 6 months and manometry data at 6 months.

The main study endpoint was the comparison of the efficacy of POEM as a treatment of achalasia between the POEM1 and POEM2 groups. The main outcome measure was the proportion of clinical success at 6 months, defined as an Eckardt score of ≤ 3 [15], in the two groups.

The secondary endpoints were the technical success and procedural, post-procedural and late POEM-related morbidity. The secondary outcome measures were the comparison between the two groups for completeness of the procedure, defined as a successful myotomy and tunnel closure, duration of the procedure adverse events that occurred intra-procedure, post-procedure or late after the procedure, and length of hospital stay.

Adverse events were graded according to the American Society for Gastrointestinal Endoscopy (ASGE) lexicon [22]. Bleeding was defined as either a two-point haemoglobin drop within 24 h or haematemesis and melaena requiring either blood cell transfusion or control endoscopy. Intra-procedure bleeding managed with haemostatic forceps was not recorded as a complication. Oesophageal perforation was defined as evidence of air or luminal contents outside of the GI tract, but, in accordance with literature [23], incidental findings of capnoperitoneum, capnothorax or capnomediastinum on post-procedure imaging and subcutaneous emphysema were not considered as adverse events. Post-procedural pain was defined as pain relieved by level 2 or 3 analgesics.

Adverse events were considered mild if they led to a prolonged hospitalization of ≤ 3 nights. They were considered moderate if they led to a prolonged hospitalization of 4–10 nights, an admission to an intensive care unit for one night or if a repeated endoscopy or an interventional radiology treatment was needed. Adverse events were considered severe if they led to a prolonged hospitalization of more than ten nights, an admission to an intensive care unit for more than one night, if a surgical treatment was required or if these events led to a permanent disability. Post-procedural adverse events occurred within 14 days and late adverse events occurred more than 14 days after the POEM.

### Statistical analyses

Quantitative variables were presented as the median and percentile (interquartile range: 25–75%). Categorical variables were presented as the number and percent of the cohort. The non-parametric Mann–Whitney test was used for continuous variables and non-parametric Pearson Chi-squared test or Fisher exact test were used for categorical variables. A *p* value < 0.05 was considered to be statistically significant. Statistical analyses were performed using JMP Pro Software, version 13.0.0 (SAS Institute Inc., Cary, NC, USA).

## Results

### Patient characteristics

From June 2015 to September 2018, 105 patients were included. The baseline characteristics of these 105 patients are listed in Table 1. The most frequent indication for POEM was type II achalasia (60%). None of the patients received opioids.

**Table 1:** Patient characteristics at baseline

	Overall population *n* = 105	POEM1 *n* = 52	POEM2 *n* = 53	*p* value
Age (years) *median* (IQR)	51.2 [38.2–64.8]	50.4 [30.5–61.1]	52.5 [42.3–67.1]	0.15
Gender (female) *n* (%)	40 (38)	22 (42)	18 (34)	0.38
Achalasia subtype *n* (%)				0.22
Type I	25 (24)	13 (25)	12 (23)	0.82
Type II	63 (60)	34 (65)	29 (55)	0.32
Type III	13 (12)	3 (6)	10 (19)	0.07
Unspecified	4 (4)	2 (4)	2 (4)	0.07
Usual weight (kg) *median* (IQR)	73 [63–84]	71 [61–81]	75 [64–85]	0.67
Weight loss (%) *median* (IQR)	5.7 [0–12.5]	6.9 [0‑13.5]	3.0 [0–12.0]	0.17
History of autoimmune disease *n* (%)	16 (15)	8 (15)	8 (16)	1.00
History of extraoesophageal neoplasia *n* (%)	9 (9)	4 (8)	5 (10)	0.73
Duration of symptoms before POEM (months) *median* (IQR)	12.2 [6.0–27.2]	3.5 [2.8–5.8]	36.6 [11.3–60.2]	0.14
Baseline Eckardt score *median* (IQR)	6 [4–8]	7 [5–9]	6 [5–8]	0.32
Dysphagia as the dominant symptom at diagnosis *n* (%)	92 (89)	44 (86)	48 (92)	0.53
Preoperative LES pressure (mmHg) *median* (IQR)^a^	30.4 [20.5–44.6]	27.7 [17.5–39.0]	33.1 [23.0–49.3]	0.10
Preoperative IRP (mmHg) *median* (IQR)^b^	21.5 [16.1–26.7]	21.5 [16.6–28.5]	19.8 [13.8–26.6]	0.54
Presence of oesophageal stasis *n* (%)	55 (53)	29 (57)	26 (50)	0.49

*IQR* interquartile range, *IRP* integrated relaxation pressure, *LES* lower oesophageal sphincter, *POEM* peroral endoscopic myotomy

^a^Data missing for 19 patients

^b^Data missing for 23 patients

The study included a total of 52 (50%) POEM1 patients and 53 (50%) POEM2 patients. Of these POEM2 patients 7 (13%) had been treated by Heller’s myotomy, 49 (92%) by pneumatic dilation and 4 (8%) by botulinum toxin injection. Several POEM2 patients had previously undergone multiple treatments: 6 (11%) patients had been treated by both Heller’s myotomy and dilation; one (2%) by both botulinum toxin injection and dilation. The average number of pneumatic dilations was 2.6 per patient.

The POEM1 and POEM2 patients were comparable according to baseline clinical and manometric characteristics. Dysphagia was the main symptom in both groups. The proportion of patients with type III achalasia tended to be higher amongst POEM2 patients but the difference was not significant (6% versus 19%; *p* = 0.07). The gastroscope could not cross the esogastric junction in 23 (22%) patients and the oesophagus lumen appeared sigmoid-like or dilated in 8 (8%) patients. Oesophageal biopsy data were available for 71 (68%) patients and none exhibited eosinophilic oesophagitis. CT scans were performed for 23 patients (22%), and no abnormalities were detected other than a sigmoid or dilated oesophagus.

### Procedural data

The rate of complete procedures was comparable between POEM1 and POEM2 patients (100% versus 96%, respectively; *p* = 0.50) (Table 2). A successful myotomy was achieved in all patients but a failure to fully close the tunnel opening using clips, due to severe submucosal fibrosis, led to a temporary placement of covered self-expanding metal stents (SEMS) in two POEM2 patients. Median procedural time was comparable between treatment-naïve patients [45 min (36–60)] and non-naïve patients [50 min (35–65); *p* = 0.16]. Length of myotomy and the number of clips were comparable between the two groups.

**Table 2:** Procedural characteristics of POEM

	Overall population *n* = 105	POEM1 *n* = 52	POEM2 *n* = 53	POEM2 *n* = 53
Complete procedure *n* (%)	103 (98)	52 (100)	51 (96)	0.50
Procedural time (minutes) *median* (IQR)	45 [36–60]	45 [36–60]	50 [35–65]	0.16
Procedural time (minutes) *median* (IQR)	10 [8–10]	10 [8–10]	9 [8–10]	0.43
Number of clips used *median* (IQR)	6 [5–7]	6 [5–7]	6 [6, 7]	0.32

*IQR* interquartile range, *POEM* peroral endoscopic myotomy

### Adverse events

Adverse events of POEM occurred in 20 patients (19%) (Table 3). Adverse events occurred intra- or post-procedure in 10 (19%) patients in both POEM1 and POEM2 groups. Of these adverse events, 13 were mild, 5 were moderate and 2 were severe, without any deaths. There was no difference between treatment-naïve and non-naïve patients. The most common adverse event was post-procedure pain which occurred in 6 (12%) POEM1 and 5 (9%) POEM2 patients (p = 0.76). No significant bleeding occurred. Two cases of mediastinitis requiring admission to an intensive care unit and classified as severe adverse events occurred: one (2%) in the POEM1 group, which was treated by surgical drainage and one (2%) in the POEM2 group, which was treated by radiological drainage, with no significant difference (*p* = 1.00). The median length of hospital stay was 3 days [3, 4] in both groups (*p* = 0.17).

**Table 3:** Procedural and post-procedural adverse events of POEM

	Overall population *n* = 105	POEM1 *n* = 52	POEM2 *n* = 53	*p* value
Total adverse events *n* (%)	20 (19)	10 (19)	10 (19)	1.00
Severe adverse events *n* (%)	2 (2)	1 (2)	1 (2)	1.00
Mediastinitis with admission to an intensive care unit	2 (2)	1 (2)	1 (2)	
Requiring surgical drainage and endoscopic stenting	1 (1)	1 (2)	0	
Requiring radiological drainage	1 (1)	0	1 (2)	
Moderate adverse events *n* (%)	5 (5)	2 (4)	3 (6)	1.00
Perforation requiring second endoscopy with clips used	2 (2)	2 (4)	0	
Mediastinitis requiring endoscopic stenting	1 (1)	0	1 (2)	
Failure to close the submucosal tunnel requiring endoscopic stenting	2 (2)	0	2 (4)	
Sequela	0	0	0	
Mild adverse events *n* (%)	13 (12)	7 (13)	6 (11)	0.78
Post-procedure pain	11 (10)	6 (12)	5 (9)	0.76
Symptomatic pneumomediastinum and pleural effusion with pneumonia (medical treatment only)	2 (2)	1 (2)	1 (2)	
Length of hospital stay (days) *median* [IQR]	3 [3, 4]	3 [3, 4]	3 [3, 4]	0.17

Regarding late adverse events, symptomatic GERD occurred in 20% of patients overall, with 11 (25%) in the POEM1 group and 7 (16%) in the POEM2 group (*p* = 0.24).

### Clinical efficacy

Data for 88 (84%) patients were available at 6 months after POEM (Table 4). Amongst these, 44 were POEM1 patients and 44 were POEM2 patients. Clinical success at 6 months was observed in 41 POEM1 patients (93%) and 37 POEM2 patients (84%) without a significant difference (*p* = 0.18). The median post-POEM Eckardt score was comparable between the two groups: one [0–2] in the POEM1 group versus one [0–2] in the POEM2 group. There was no significant difference in the post-POEM IRP and LES resting pressure between the two groups (Table 4). The clinical efficacy between the different subgroups of first-line treatment in POEM2 patients is shown in the Supplementary materials section. In patients with dilated and/or sigmoid oesophagus, clinical success at 6 months occurred in 5/7 patients (1 missing data). Clinical efficacy at 12 months was only available for 51 patients (49%), without any difference between the 2 groups (81% in POEM1 and 80% in POEM2) (Supplementary material).

**Table 4:** Outcomes and late adverse events at 6 months after POEM

	Overall population *n* = 88	POEM1 *n* = 44	POEM2 *n* = 44	*p* value
Clinical success (Eckardt score ≤ 3) *n* (%)	78 (89)	41 (93)	37 (84)	0.18
Post-operative LES pressure *median* [IQR]^a^	12.8 [10.0–20.4]	11.9 [10.5–19.4]	15.2 [10.0–20.5]	0.73
Post-operative IRP *median* [IQR]^b^	7.3 [4.7–11.3]	7.1 [4.6–9.8]	7.7 [4.7–13.2]	0.95
GERD symptoms *n* (%)^*c*^	18 (20)	11 (25)	7 (16)	0.24
Proton pump inhibitors used *n* (%)^d^	19 (22)	10 (23)	9 (20)	0.66

*GERD* gastro-oesophageal reflux disease, *IQR* interquartile range, *IRP* integrated relaxation pressure, *LES* lower oesophageal sphincter, *POEM* peroral endoscopic myotomy

^a^Data analysed in 63 patients

^b^Data analysed in 61 patients

^c^Data analysed in 84 patients

^d^Data analysed in 83 patients

## Discussion

The present work demonstrates that the efficacy, feasibility and safety of POEM are comparable between treatment-naïve and non-naïve patients with achalasia. Firstly, the clinical success rate at 6 months was at 93% in POEM1 patients and 84% in POEM2 patients without a significant difference between the two groups. Secondly, oesophageal myotomy was successfully performed in all patients; however, two POEM2 patients experienced a failure of tunnel closure by clips, which was successfully treated by oesophageal stenting. Thirdly, the occurrence of procedural, post-procedural and late adverse events and the length of hospital stay was not significantly different between the groups. Together, these results suggest that POEM could be proposed as a second-line treatment for patients with persistent symptoms after surgical or endoscopic treatment of achalasia.

Other studies have compared the feasibility and the outcomes of POEM between treatment-naïve and non-naïve patients with achalasia [17, 24–27]. Indeed, a previous study compared the efficacy, feasibility and adverse event rates in three groups: treatment-naïve patients, patients with a history of submucosal injections or dilations and patients with sigmoid oesophagus or history of surgical treatment. In this latter group, the operative time was longer; however, the clinical success was comparable in all groups [24]. In our study, the imbalance between the numbers of patients who have previously been treated by dilation (*n* = 49), Heller’s myotomy (*n* = 7) and botulinum toxin injection (*n* = 4) prevented us from performing statistical analyses on the efficacy of POEM in different subgroups. Likewise, we could not compare the results of POEM in patients with sigmoid oesophagus to patients without dilated oesophagus. Results of a recent meta-analysis conducted mostly in Asian centres on 487 patients which investigated the efficacy of POEM as a second-line treatment are also in accordance with our results [25]. Recent guidelines from the American Society for Gastrointestinal Endoscopy (ASGE) and European Society for Gastrointestinal Endoscopy (ESGE) [3, 5] suggest that in case of recurrence after treatment either pneumatic dilations, Heller’s myotomy or POEM can be performed. However, only low level of evidence of their efficacy exists due to the small number of studies. In this context, this case-controlled study brings new evidence of the relevance of second-line POEM treatment.

Overall, the rates of severe adverse events and clinical success at 6 months were 2% and 89%, which are, respectively, higher and lower than the rates reported by large series [14, 28, 29]. However, no death or sequela was noted in our patients. The rate of clinical success at 12 months was 80% (Supplementary material) but is subject to bias, as only 49% of patients could be evaluated in this retrospective work. Regarding efficacy, our results are in line with a previous prospective international European study [30] reporting 82% efficacy at 1 year, but are lower than those reported by a recent Japanese multicentre prospective study reporting 97% efficacy at 1 year [28]. However, this study was performed in very expert centres with a specific technique that included a double endoscope procedure and indocyanine green injection in the submucosa. Other hypotheses can explain our mixed results. Firstly, all consecutive patients were included regardless of age, comorbidities or the severity of the disease, providing real-life conditions. In our series, 17 patients (16%) over 70 years of age were included, whilst it has been reported that the rate of adverse events is slightly higher in elderly patients [31]. In addition, the proportion of associated diseases, such as cancer (9%) and autoimmune disease (15%), was high in our study. Secondly, all POEM cases performed by the operator, including the first cases, were reported. This might have affected the overall results, although the distribution of adverse events and the distribution of patients by group were homogeneous over time in our study (data not shown). Indeed, in the literature the results on the efficacy and morbidity depend on the number of interventions performed with an estimated number of procedures required to achieve proficiency varying between 15 [32, 33] and 100 [34]. Thirdly, the overall results in our series might also have been affected by the complexity of some of the patients’ previous therapies and the complexity of the disease. For instance, our series had a large proportion of type III achalasia, in 19% of non-naïve and 6% of treatment-naïve patients. It is worth noting that type III achalasia seems to be consistently more difficult to treat with the usual techniques [35–37] and both ESGE and ASGE guidelines suggest that in the case of type III achalasia, POEM should be attempted as a first-line treatment [3, 38]. Another interesting point is that fibrosis caused by previous treatments led to a failure of tunnel closure using clips in two patients, which was successfully treated using oesophageal stenting. One patient had undergone 3 oesophageal dilations and the other had been treated with botulinum toxin injection. The small number of patients in the different subgroups prevented us from performing an analysis of the adverse events according to the type of previous treatment. Other series have also reported comparable difficult cases [39] after multiple submucosal injections and dilations with the need of stent placement and prolonged hospitalization. Endoscopists should be prepared to manage these challenging cases and keep in mind the difficulty of the procedure. In addition, it should be noted that amongst the patients in symptomatic relapse at 6 months, four had a second successful POEM, since a repeat POEM appears to be a safe and effective solution [36]. Future studies should address the question of the long-term efficacy of POEM as a second-line therapy for the treatment of achalasia.

The median length of hospital stay was similar in the 2 groups. Although it may seem long compared with previous studies [32, 33], it is close to the length of stay observed in other series [14, 29]. It is worth noting that this series reflects the first cases of a single operator; the length of stay was long as a precaution. Also, patients often spent the night before POEM in the hospital because they did not always live near the tertiary centre where POEM was performed. They usually left the hospital the day after the procedure or the following day, depending on their tolerance.

In conclusion, POEM appears to be an effective, feasible and safe approach for patients who have undergone either endoscopic or surgical treatment for achalasia and can thus be offered as a second-therapeutic line. Prospective long-term follow-up to confirm these data is required.

## Supplementary Information

Below is the link to the electronic supplementary material.

Supplementary file1 (DOCX 13 KB)

## References

[1] Boeckxstaens GE, Zaninotto G, Richter JE (2014). Achalasia. Lancet.

[2] Kahrilas PJ, Bredenoord AJ, Fox M, Gyawali CP, Roman S, Smout AJPM, Pandolfino JE, International High Resolution Manometry Working Group (2015). The Chicago classification of esophageal motility disorders, v3.0. Neurogastroenterol Motil.

[3] Khashab MA, Vela MF, Thosani N, Agrawal D, Buxbaum JL, Abbas Fehmi SM, Fishman DS, Gurudu SR, Jamil LH, Jue TL, Kannadath BS, Law JK, Lee JK, Naveed M, Qumseya BJ, Sawhney MS, Yang J, Wani S (2020). ASGE guideline on the management of achalasia. Gastrointest Endosc.

[4] Schlottmann F, Luckett DJ, Fine J, Shaheen NJ, Patti MG (2018). Laparoscopic Heller myotomy versus peroral endoscopic myotomy (POEM) for achalasia: a systematic review and meta-analysis. Ann Surg.

[5] Oude Nijhuis R, Zaninotto G, Roman S, Boeckxstaens GE, Fockens P, Langendam MW, Plumb AA, Smout A, Targarona EM, Trukhmanov AS, Weusten B, Bredenoord AJ (2020). European guidelines on achalasia: United European gastroenterology and European society of neurogastroenterology and motility recommendations. United Eur Gastroenterol J.

[6] Moonen A, Annese V, Belmans A, Bredenoord AJ, Bruley des Varannes S, Costantini M, Dousset B, Elizalde JI, Fumagalli U, Gaudric M, Merla A, Smout AJ, Tack J, Zaninotto G, Busch OR, Boeckxstaens GE (2016). Long-term results of the European achalasia trial: a multicentrerandomised controlled trial comparing pneumatic dilation versus laparoscopic Heller myotomy. Gut.

[7] Boeckxstaens GE, Annese V, Varannes SB, Chaussade S, Costantini M, Cuttitta A, Elizalde JI, Fumagalli U, Gaudric M, Rohof WO, Smout AJ, Tack J, Zwinderman AH, Zaninotto G, Busch OR, Investigators EAT (2011). Pneumatic dilation versus laparoscopic Heller’s myotomy for idiopathic achalasia. N Engl J Med.

[8] Loviscek MF, Wright AS, Hinojosa MW, Petersen R, Pajitnov D, Oelschlager BK, Pellegrini CA (2013). Recurrent dysphagia after Heller myotomy: is esophagectomy always the answer?. J Am Coll Surg.

[9] Pallati PK, Mittal SK (2011). Operative interventions for failed Heller myotomy: a single institution experience. Am Surg.

[10] Kumbhari V, Behary J, Szczesniak M, Zhang T, Cook IJ (2013). Efficacy and safety of pneumatic dilatation for achalasia in the treatment of post-myotomy symptom relapse. Am J Gastroenterol.

[11] Legros L, Ropert A, Brochard C, Bouguen G, Pagenault M, Siproudhis L, Bretagne J-F (2014). Long-term results of pneumatic dilatation for relapsing symptoms of achalasia after Heller myotomy. Neurogastroenterol Motil.

[12] Inoue H, Minami H, Kobayashi Y, Sato Y, Kaga M, Suzuki M, Satodate H, Odaka N, Itoh H, Kudo S (2010). Peroral endoscopic myotomy (POEM) for esophageal achalasia. Endoscopy.

[13] Akintoye E, Kumar N, Obaitan I, Alayo QA, Thompson CC (2016). Peroral endoscopic myotomy: a meta-analysis. Endoscopy.

[14] Inoue H, Sato H, Ikeda H, Onimaru M, Sato C, Minami H, Yokomichi H, Kobayashi Y, Grimes KL, Kudo S (2015). Per-oral endoscopic myotomy: a series of 500 patients. J Am Coll Surg.

[15] Teitelbaum EN, Dunst CM, Reavis KM, Sharata AM, Ward MA, DeMeester SR, Swanström LL (2018). Clinical outcomes five years after POEM for treatment of primary esophageal motility disorders. Surg Endosc.

[16] Ponds FA, Fockens P, Lei A, Neuhaus H, Beyna T, Kandler J, Frieling T, Chiu PWY, Wu JCY, Wong VWY, Costamagna G, Familiari P, Kahrilas PJ, Pandolfino JE, Smout AJPM, Bredenoord AJ (2019). Effect of peroral endoscopic myotomy vs pneumatic dilation on symptom severity and treatment outcomes among treatment-naive patients with achalasia: a randomized clinical trial. JAMA.

[17] Kristensen HØ, Kirkegård J, Kjær DW, Mortensen FV, Kunda R, Bjerregaard NC (2017). Long-term outcome of peroral endoscopic myotomy for esophageal achalasia in patients with previous Heller myotomy. Surg Endosc.

[18] Zhou PH, Li QL, Yao LQ, Xu MD, Chen WF, Cai MY, Hu JW, Li L, Zhang YQ, Zhong YS, Ma LL, Qin WZ, Cui Z (2013). Peroral endoscopic remyotomy for failed Heller myotomy: a prospective single-center study. Endoscopy.

[19] Ling T, Guo H, Zou X (2014). Effect of peroral endoscopic myotomy in achalasia patients with failure of prior pneumatic dilation: a prospective case-control study. J Gastroenterol Hepatol.

[20] Eckardt VF, Aignherr C, Bernhard G (1992). Predictors of outcome in patients with achalasia treated by pneumatic dilation. Gastroenterology.

[21] Daabiss M (2011). American society of anaesthesiologists physical status classification. Indian J Anaesth.

[22] Cotton PB, Eisen GM, Aabakken L, Baron TH, Hutter MM, Jacobson BC, Mergener K, Nemcek A, Petersen BT, Petrini JL, Pike IM, Rabeneck L, Romagnuolo J, Vargo JJ (2010). A lexicon for endoscopic adverse events: report of an ASGE workshop. Gastrointest Endosc.

[23] Haito-Chavez Y, Inoue H, Beard KW, Draganov PV, Ujiki M, Rahden BHA, Desai PN, Pioche M, Hayee B, Haji A, Saxena P, Reavis K, Onimaru M, Balassone V, Nakamura J, Hata Y, Yang D, Pannu D, Abbas A, Perbtani YB, Patel LY, Filser J, Roman S, Rivory J, Mion F, Ponchon T, Perretta S, Wong V, Maselli R, Ngamruengphong S, Chen Y-I, Bukhari M, Hajiyeva G, Ismail A, Pieratti R, Kumbhari V, Galdos-Cardenas G, Repici A, Khashab MA (2017). Comprehensive analysis of adverse events associated with per oral endoscopic myotomy in 1826 patients: an international multicenter study. Am J Gastroenterol.

[24] Louie BE, Schneider AM, Schembre DB, Aye RW (2017). Impact of prior interventions on outcomes during per oral endoscopic myotomy. Surg Endosc.

[25] Hashimoto R, Inoue H, Shimamura Y, Sakuraba A, Tomizawa Y (2020). Per oral endoscopic myotomy as salvage therapy in patients with achalasia refractory to endoscopic or surgical therapy is technically feasible and safe: systematic review and meta-analysis. Dig Endosc.

[26] Nabi Z, Ramchandani M, Chavan R, Tandan M, Kalapala R, Darisetty S, Lakhtakia S, Rao GV, Reddy DN (2018). Peroral endoscopic myotomy in treatment-naïve achalasia patients versus prior treatment failure cases. Endoscopy.

[27] Sharata A, Kurian AA, Dunst CM, Bhayani NH, Reavis KM, Swanström LL (2013). Peroral endoscopic myotomy (POEM) is safe and effective in the setting of prior endoscopic intervention. J Gastrointest Surg.

[28] Shiwaku H, Inoue H, Yamashita K, Ohmiya T, Beppu R, Nakashima R, Takeno S, Sasaki T, Nimura S, Yamashita Y (2016). Peroral endoscopic myotomy for esophageal achalasia: outcomes of the first over 100 patients with short-term follow-up. Surg Endosc.

[29] Familiari P, Gigante G, Marchese M, Boskoski I, Tringali A, Perri V, Costamagna G (2016). Peroral endoscopic myotomy for esophageal achalasia: outcomes of the first 100 patients with short-term follow-up. Ann Surg.

[30] Von Renteln D, Fuchs K-H, Fockens P, Bauerfeind P, Vassiliou MC, Werner YB, Fried G, Breithaupt W, Heinrich H, Bredenoord AJ, Kersten JF, Verlaan T, Trevisonno M, Rösch T (2013). Peroral endoscopic myotomy for the treatment of achalasia: an international prospective multicenter study. Gastroenterology.

[31] Chen Y-I, Inoue H, Ujiki M, Draganov PV, Colavita P, Mion F, Romanelli J, Chiu P, Balassone V, Patel L, Abbas A, Yang D, Dunst C, Pioche M, Roman S, Rivory J, Ponchon T, Desilets D, Maselli R, Onimaru M, Nakamura J, Hata Y, Hajiyeva G, Ismail A, Ngamruengphong S, Bukhari M, Chavez YH, Kumbhari V, Repici A, Khashab MA (2018). An international multicenter study evaluating the clinical efficacy and safety of per-oral endoscopic myotomy in octogenarians. Gastrointest Endosc.

[32] El Zein M, Kumbhari V, Ngamruengphong S, Carson KA, Stein E, Tieu A, Chaveze Y, Ismail A, Dhalla S, Clarke J, Kalloo A, Canto MI, Khashab MA (2016). Learning curve for peroral endoscopic myotomy. Endosc Int Open.

[33] Hungness ES, Sternbach JM, Teitelbaum EN, Kahrilas PJ, Pandolfino JE, Soper NJ (2016). Per-oral endoscopic myotomy (POEM) after the learning curve: durable long-term results with a low complication rate. Ann Surg.

[34] Liu Z, Zhang X, Zhang W, Zhang Y, Chen W, Qin W, Hu J, Cai M, Zhou P, Li Q (2018). Comprehensive evaluation of the learning curve for peroral endoscopic myotomy. Clin Gastroenterol Hepatol.

[35] Rohof WO, Salvador R, Annese V, Bruley des Varannes S, Chaussade S, Costantini M, Elizalde JI, Gaudric M, Smout AJ, Tack J, Busch OR, Zaninotto G, Boeckxstaens GE (2013). Outcomes of treatment for achalasia depend on manometric subtype. Gastroenterology.

[36] Tyberg A, Seewald S, Sharaiha RZ, Martinez G, Desai AP, Kumta NA, Lambroza A, Sethi A, Reavis KM, DeRoche K, Gaidhane M, Talbot M, Saxena P, Zamarripa F, Barret M, Eleftheriadis N, Balassone V, Inoue H, Kahaleh M (2017). A multicenter international registry of redo per-oral endoscopic myotomy (POEM) after failed POEM. Gastrointest Endosc.

[37] Andolfi C, Fisichella PM (2019). Meta-analysis of clinical outcome after treatment for achalasia based on manometric subtypes. Br J Surg.

[38] Weusten B, Bisschops R, Coron E, Dinis-Ribeiro M, Dumonceau J-M, Esteban J-M, Hassan C, Pech O, Repici A, Bergman J, di Pietro M (2017). Endoscopic management of Barrett’s esophagus: European society of gastrointestinal endoscopy (ESGE) position statement. Endoscopy.

[39] Orenstein SB, Raigani S, Wu YV, Pauli EM, Phillips MS, Ponsky JL, Marks JM (2015). Peroral endoscopic myotomy (POEM) leads to similar results in patients with and without prior endoscopic or surgical therapy. Surg Endosc.

