# Supplementary material for: Peroral endoscopic myotomy: is it better to perform it in naive patients or as second-line therapy? Results of an open-label-controlled study in 105 patients
Source: Surg Endosc. 2023 Jan 20;37(5):3760–8. doi: 10.1007/s00464-021-08767-6 (PMC10156842; doi:10.1007/s00464-021-08767-6)
Supplement: Supplementary file 1 — Supplementary file1 (DOCX 13 KB) [file 464_2021_8767_MOESM1_ESM.docx]

**Table S1: Outcomes and late adverse events at 12 months after POEM**

|  | **Overall population**  **n=51** | **POEM1**  **n=21** | **POEM2**  **n=30** | **p-value** |
| --- | --- | --- | --- | --- |
| **Clinical success (Eckardt score ≤3) *n (%)*** | 41(80) | 17(81) | 24(80) | ns |
| **GERD symptoms**  ***n (%)^a^*** | 15(30) | 4(19) | 11(38) | ns |
| **Proton pump inhibitors used**  ***n (%)^b^*** | 17(35) | 5(25) | 12(41) | ns |

^a^data analysed in 50 patients; ^b^ data analysed in 49 patients

**Table S2: Outcomes in POEM1 and POEM2 subgroups at 6 months after POEM**

|  | Dilations ± botulinum toxin injection  n=36 | Heller’s myotomy after dilation  n=5 | Botulinum toxin injection only  n=3 |
| --- | --- | --- | --- |
| **Clinical success (Eckardt score ≤3) *n(%)*** | 32(89) | 4(80) | 1(33) |
| **Post-operative LES pressure *median [IQR]*** | 15.5 [9.4-20.8] **^a^** | 12.8 [11-15.2] | 20.5 [7.3-31.6] |
| **Post-operative IRP *median [IQR]*** | 7.7 [4.8-14.6] **^b^** | 6.1 [4.6-11.4] | 10.6 [1.9-11.2] |

**^a^** data analysed in 24 patients; **^b^**data analysed in 23 patients
